# Supplementary material for: Decoupling light absorption and carrier transport via heterogeneous doping in Ta3N5 thin film photoanode
Source: Nat Commun. 2022 Dec 15;13:7769. doi: 10.1038/s41467-022-35538-1 (PMC9755297; doi:10.1038/s41467-022-35538-1)
Supplement: Supplementary file 1 — Supplementary Information [file 41467_2022_35538_MOESM1_ESM.pdf]

Supplementary Information

Decoupling light absorption and carrier transport via heterogeneous doping in Ta_3N_5 thin film photoanode

Yequan Xiao,[1] Zeyu Fan,[1] Mamiko Nakabayashi,[2] Qiaoqiao Li,[3] Liujiang Zhou,[3] Qian Wang,[4,5] Changli Li,[6] Naoya Shibata,[2] Kazunari Domen,[7,8] & Yanbo Li[1,*]

[1]Institute of Fundamental and Frontier Sciences, University of Electronic Science and Technology of China, Chengdu 610054, China.

[2]Institute of Engineering Innovation, The University of Tokyo, Tokyo 113-8656, Japan.

[3]School of Physics, University of Electronic Science and Technology of China, Chengdu 610054, China.

[4]Graduate School of Engineering, Nagoya University, Nagoya 464-8603, Japan

[5]Institute for Advanced Research, Nagoya University, Nagoya 464-8601, Japan

[6]School of Materials, Sun Yat-sen University, Guangzhou 510275, China

[7]Office of University Professors, The University of Tokyo, Tokyo 113-8656, Japan

[8]Research Initiative for Supra-Materials (RISM), Shinshu University, Nagano 380-8553, Japan

[*]E-mail: yanboli@uestc.edu.cn

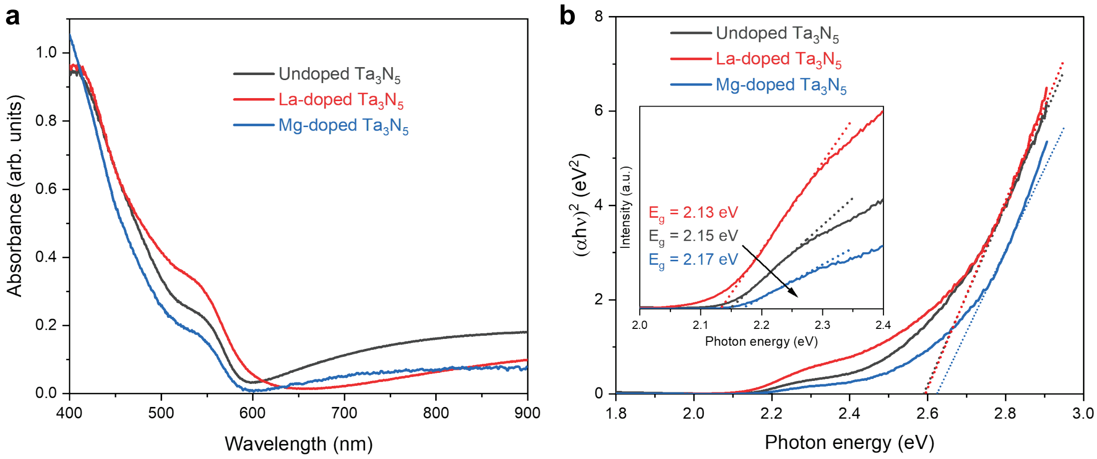

Supplementary Fig. 1 | Optical absorption properties of undoped, La-doped and Mg-doped Ta_3N_5 films on quartz substrates. a, UV-vis absorption spectra. The thickness of all films is about 100 nm. The Mg/Ta concentration in the Mg-doped Ta_3N_5 thin film was 13.5% estimated from XPS results (Supplementary Fig. 6). Compared with the undoped Ta_3N_5 film, the above-bandgap light absorption is weakened in the Mg-doped sample, while that of the La-doped sample is notably enhanced in the range of 480-590 nm. **b**, Tauc plots of UV-vis absorption spectra. *α*, absorption coefficient; *h*, Planck's constant; *v*, photon's frequency. The dotted lines show the extrapolation of the linear portion of the absorption edges. Mg doping causes an increase in the optical bandgap, while La doping narrows the bandgap of Ta_3N_5 films.

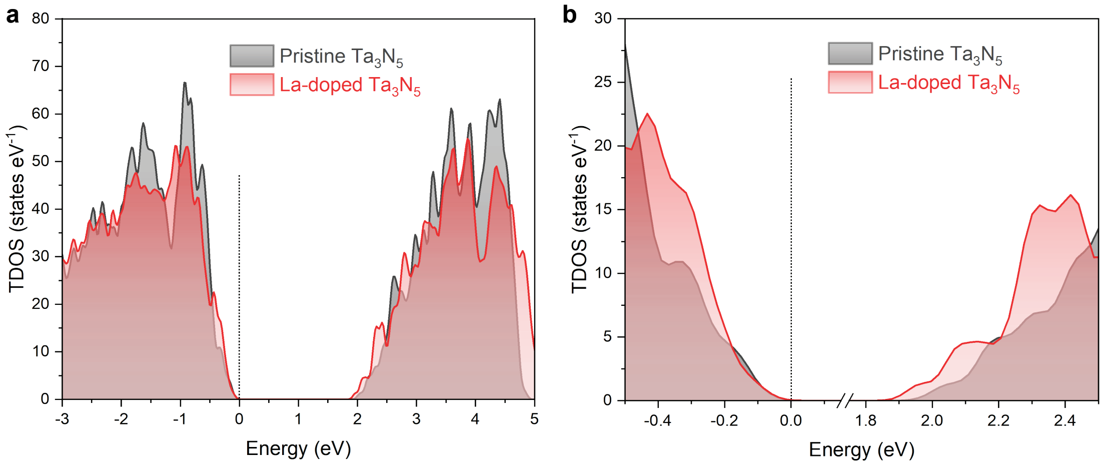

Supplementary Fig. 2 | Calculated total density of states (TDOS). a, TDOS for pristine and La-doped Ta_3N_5 without intrinsic defects. **b,** TDOS near the conduction band minimum (CBM) and valence band maximum (VBM). The density of states around Fermi level are enhanced by La doping and more delocalized orbital distribution are observed in the vicinity of CBM and VBM, which are stemming from the increased hybridization between La and N orbitals in La-doped system.

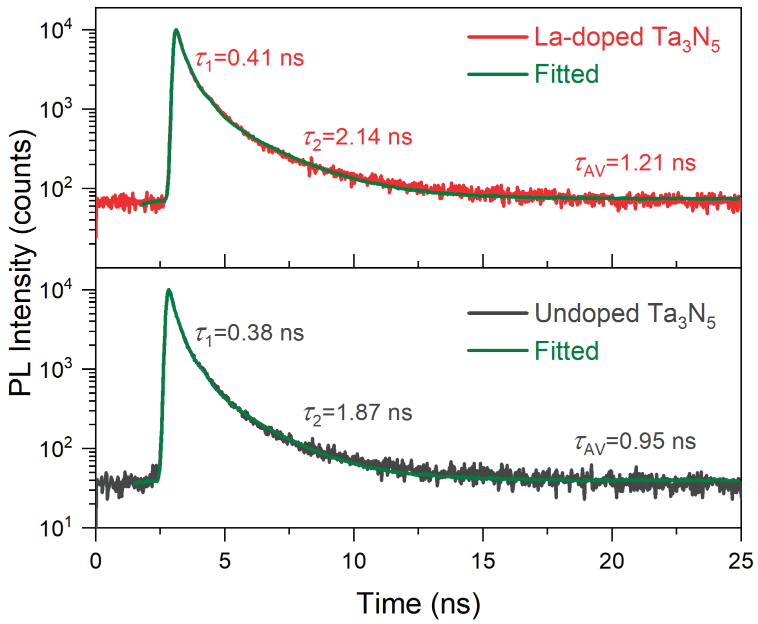

Supplementary Fig. 3 | Low-temperature TRPL decay profile for undoped and La-doped Ta_3N_5 thin films measured at 10 K under 510 nm laser excitation. The TRPL decay curves were acquired at 610 nm with 20 nm bandwidth under the excitation of a 510 nm picosecond laser pulsed at a repetition rate of 40 MHz. The system instrument response function was measured and used as the reference to fit the decay with a numerical re-convolution algorithm. Both decay curves can be well-fitted using a biexponential decay model. The intensity-weighted average lifetime (τ_{AV}) is calculated by: $\tau_{AV} = f_1\tau_1 + f_2\tau_2$, where f_1 and f_2 are the fractional intensities of the decay channels with lifetimes τ_1 (defect-related recombination) and τ_2 (the electron-hole recombination from conduction to valence band), respectively. The lifetime of the fast decay component (τ_1) was similar for both undoped Ta_3N_5 (0.38 ns) and La-doped Ta_3N_5 (0.41 ns) films. However, the fractional intensity of the fast component (f_1) was less for La-doped Ta_3N_5 (53.7%) than for undoped Ta_3N_5 (62.1%). The lifetime of the slow component (τ_2) was longer for La-doped Ta_3N_5 (2.15 ns, f_1 = 46.3%) than for undoped Ta_3N_5 (1.87 ns, f_2 = 37.9%).

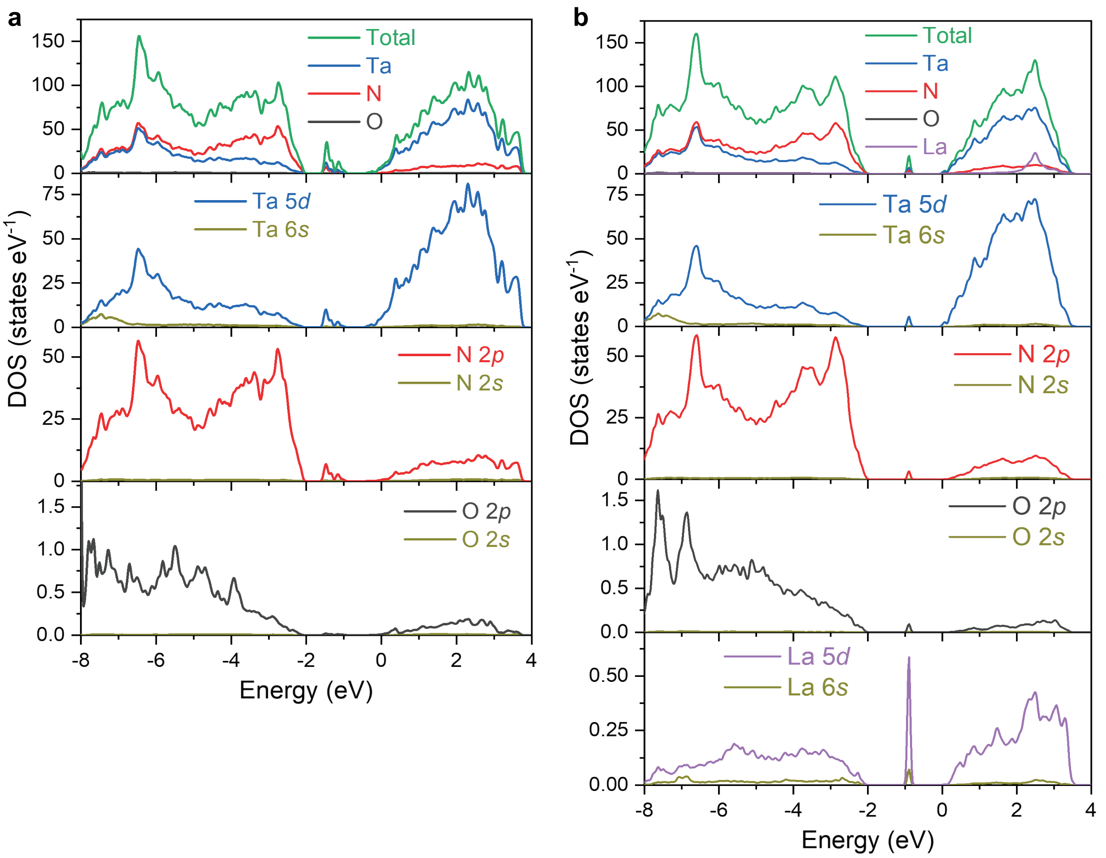

Supplementary Fig. 4 | Calculated densities of states (DOS) of undoped Ta_3N_5 (a) and La-doped Ta_3N_5 (b) with intrinsic O_N, V_N, and Ta^{3+} defects. The intermediate states mainly consist of Ta 5*d*, N 2*p* orbitals in undoped Ta_3N_5. Upon doping La into Ta_3N_5 lattice, the intermediate states mostly originate from Ta 5*d*, N 2*p* orbitals, and a small amount of La 5*d* orbital. Also, the density of intermediate states is significantly reduced in La-doped Ta_3N_5.

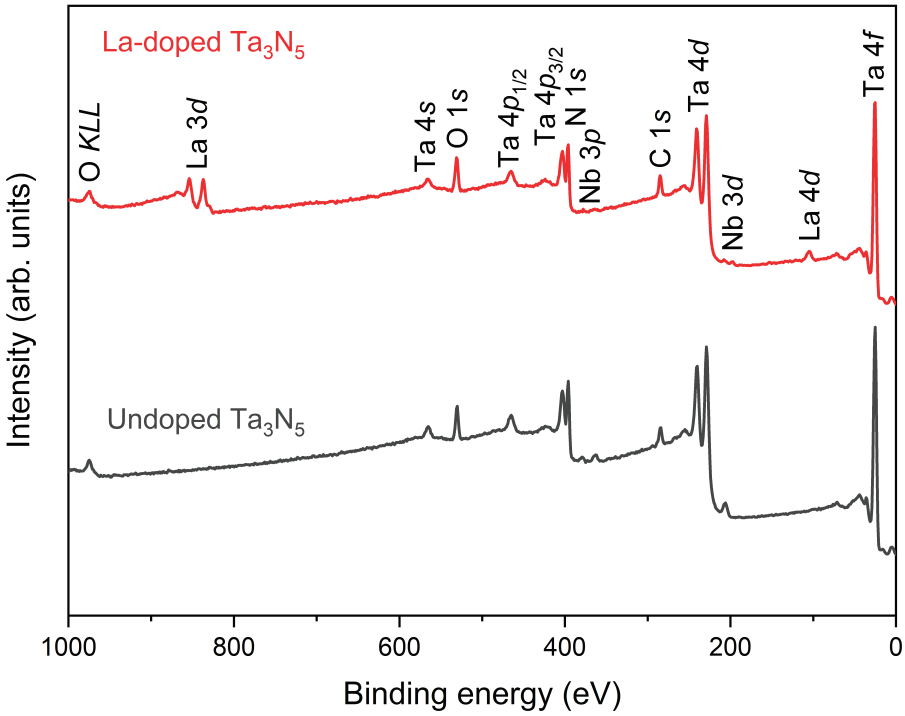

Supplementary Fig. 5 | XPS survey spectra for undoped and La-doped Ta_3N_5 films. Charging shift compensation was made for both curves using C 1s at 284.8 eV as the reference. Compared with undoped Ta_3N_5 sample, the La-doped Ta_3N_5 sample clearly revealed peaks from La, in addition to those from Ta, N, O, and C.

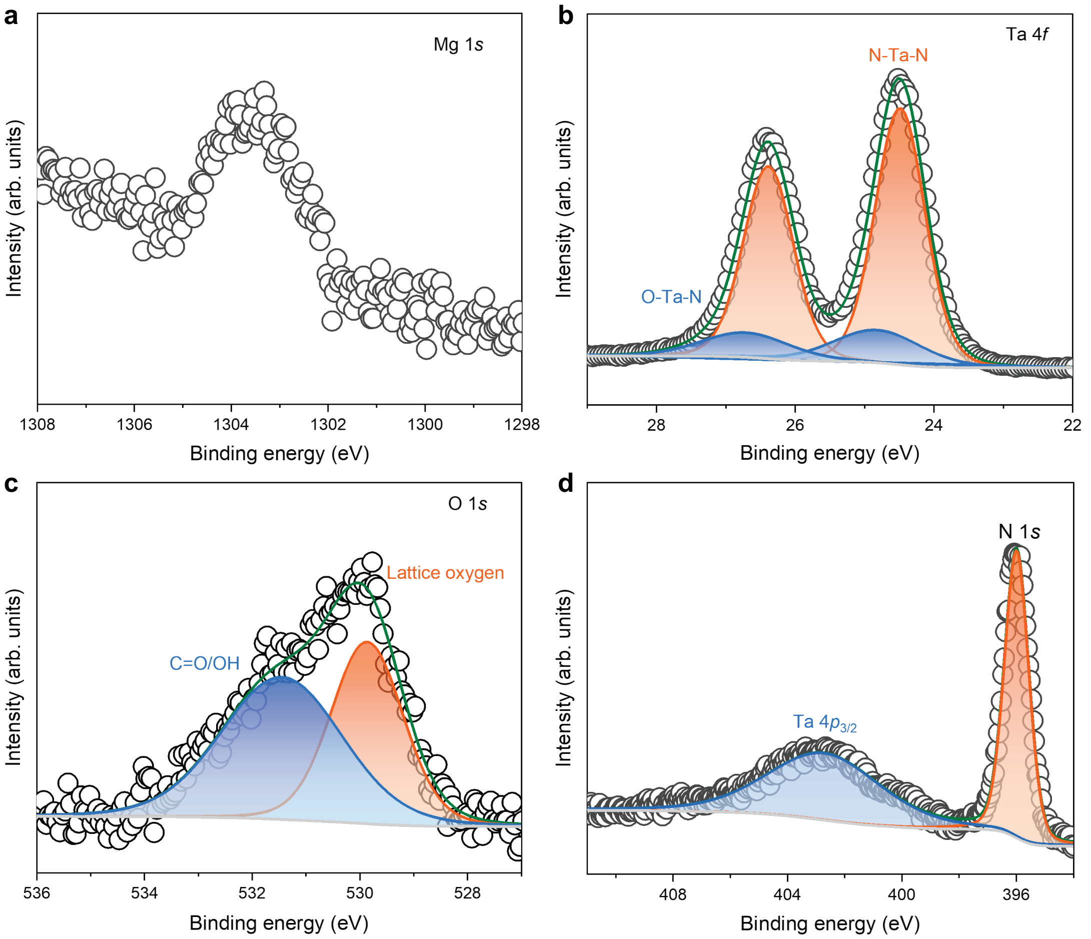

Supplementary Fig. 6 | XPS core-level spectra of Mg-doped Ta_3N_5. a, Mg 1*s* peak. **b**, Ta 4*f* peak. **c**, O 1*s* peak. **d**, N 1*s* peak. Quantitative XPS analyses showed that the Mg/Ta concentration was 13.50%, and the N/Ta and O/Ta atom ratios were 1.62 and 0.30, respectively. These results are in accordance with our previous studies, which suggested that Mg doping can efficiently reduce deep traps created by nitrogen vacancies and increase shallow donors generated by oxygen impurities in Ta_3N_5 (*Ref. 1*). Combining XPS analysis of undoped and La-doped Ta_3N_5 samples (Fig. 2), it was found that Mg doping increased both oxygen and nitrogen contents in Ta_3N_5 films, while La doping had a greater effect on the increase in oxygen content.

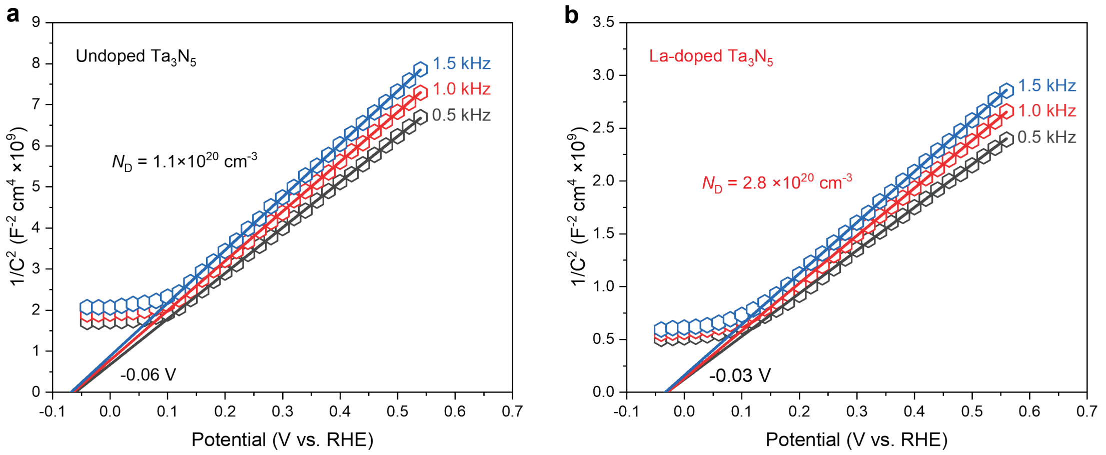

Supplementary Fig. 7 | Mott-Schottky plots of undoped Ta_3N_5 (a) and La-doped Ta_3N_5 (b) films. The data was obtained by performing a potential scan in the anodic direction under frequencies of 0.5, 1.0 and 1.5 kHz with an AC amplitude of 15 mV in 1 M KOH electrolyte at pH 13.6 under the dark conditions. Before the measurement, each sample was dipped in a mixed etchant of HF: HNO_3: H_2O (1:2:7 in v/v) for 20 s to prepare a fresh surface, and then rinsed with deionized water. The electrolyte was stirred and purged with Ar for 20 min. Cyclic voltammetry was performed repeatedly to clean the surface of the electrode. The lines show the fitting of the linear regions of the Mott-Schottky plots. From the interceptions of the lines with the *x*-axis, the flat-band potentials of the samples were obtained, ca.-0.06 V versus RHE for the undoped Ta_3N_5 film and ca.-0.03 V versus RHE for the La-dope Ta_3N_5 film. The flat-band potential of the La-dope Ta_3N_5 are very similar to the undoped Ta_3N_5, which coincides with the similar onset potential for the two photoanodes in Fig. 1f. From the slope (k_S) of the lines, the carrier concentration (N_D) in the samples could be estimated from the equation $N_D = 2/(e\varepsilon_0\varepsilon_r k_S)$, where e is electron charge, ε_0 is vacuum permittivity, and ε_r is the dielectric constant of Ta_3N_5 (~110). The carrier concentration at the frequency of 1.0 kHz in undoped and La-dope Ta_3N_5 films are estimated to be 1.1×10^{20} cm^{-3} and 2.8×10^{20} cm^{-3}, respectively.

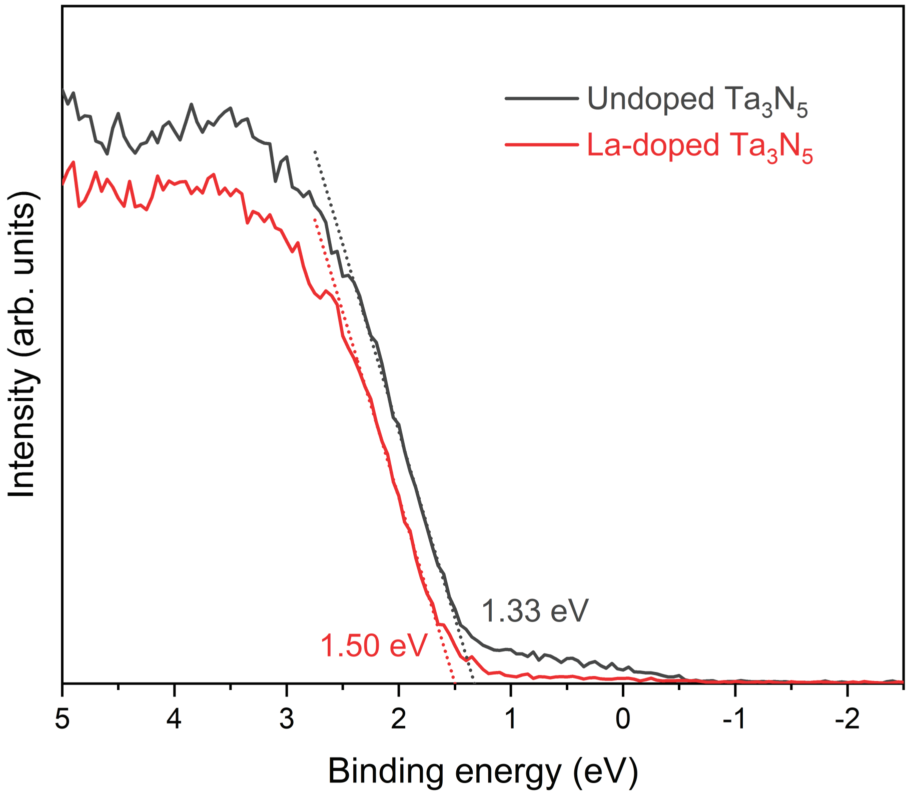

Supplementary Fig. 8 | XPS valence band spectra of undoped and La-doped Ta_3N_5 films.

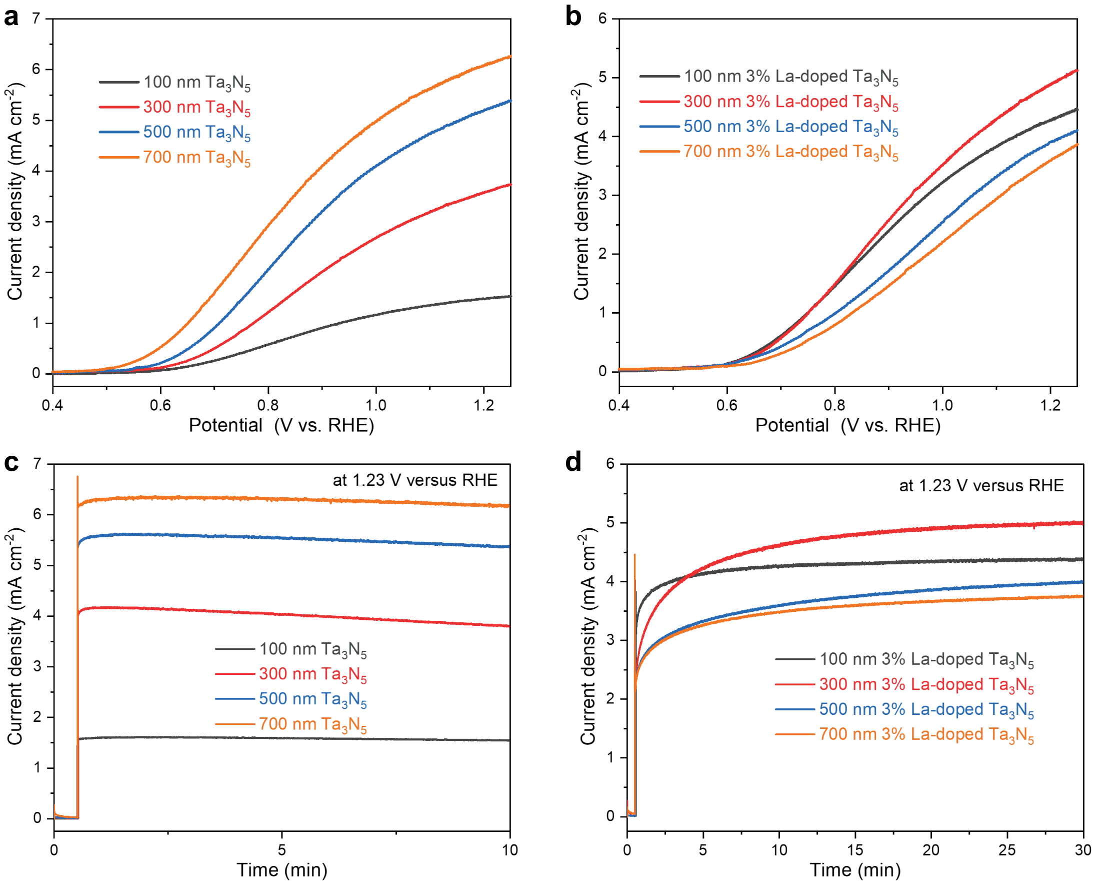

Supplementary Fig. 9 | Thickness-dependent PEC water splitting properties for undoped and La-doped Ta_3N_5 thin film photoanodes. All samples were modified with NiCoFe-B_i cocatalyst and tested under AM 1.5G simulated sunlight in 1 M KOH**.** **a**, *J*-V curves of undoped Ta_3N_5 photoanodes. **b**, *J-V* curves of La-doped Ta_3N_5 photoanodes. **c**, Steady-state photocurrent of undoped Ta_3N_5 photoanode at an applied potential of 1.23 V versus RHE for 10 min. **d**, Steady-state photocurrent of La-doped Ta_3N_5 photoanode at an applied potential of 1.23 V versus RHE for 30 min.

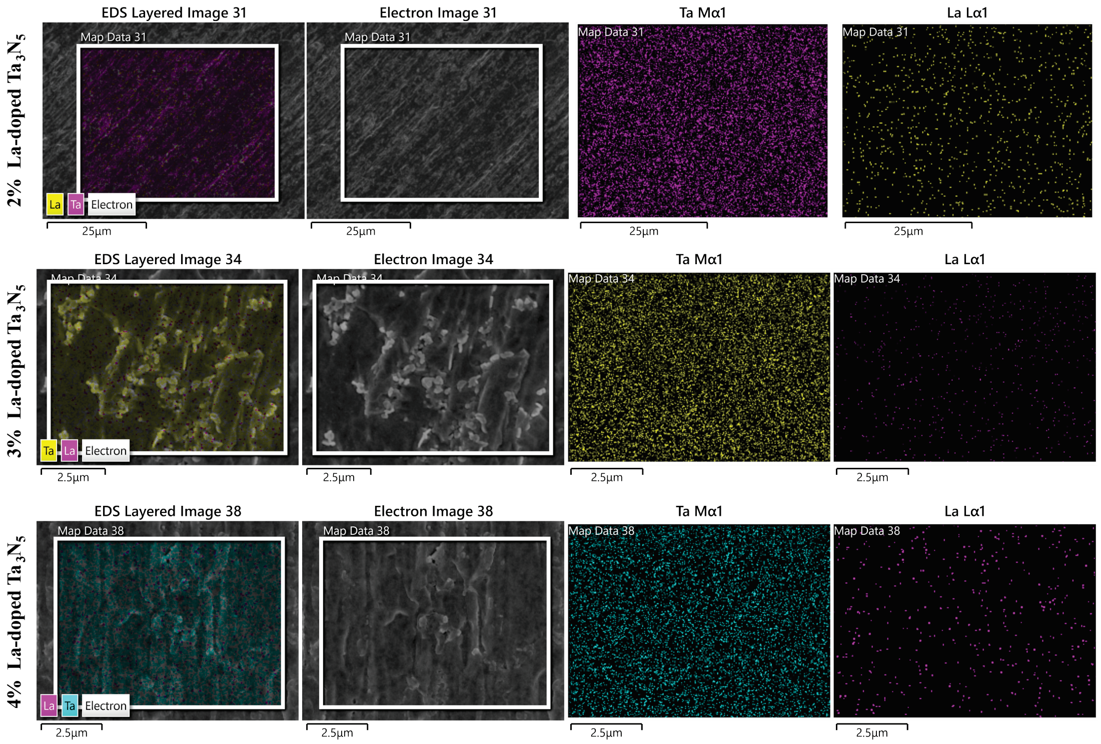

Supplementary Fig. 10 | SEM-EDS elemental mapping of La and Ta in Ta_3N_5 films with different La doping concentrations.

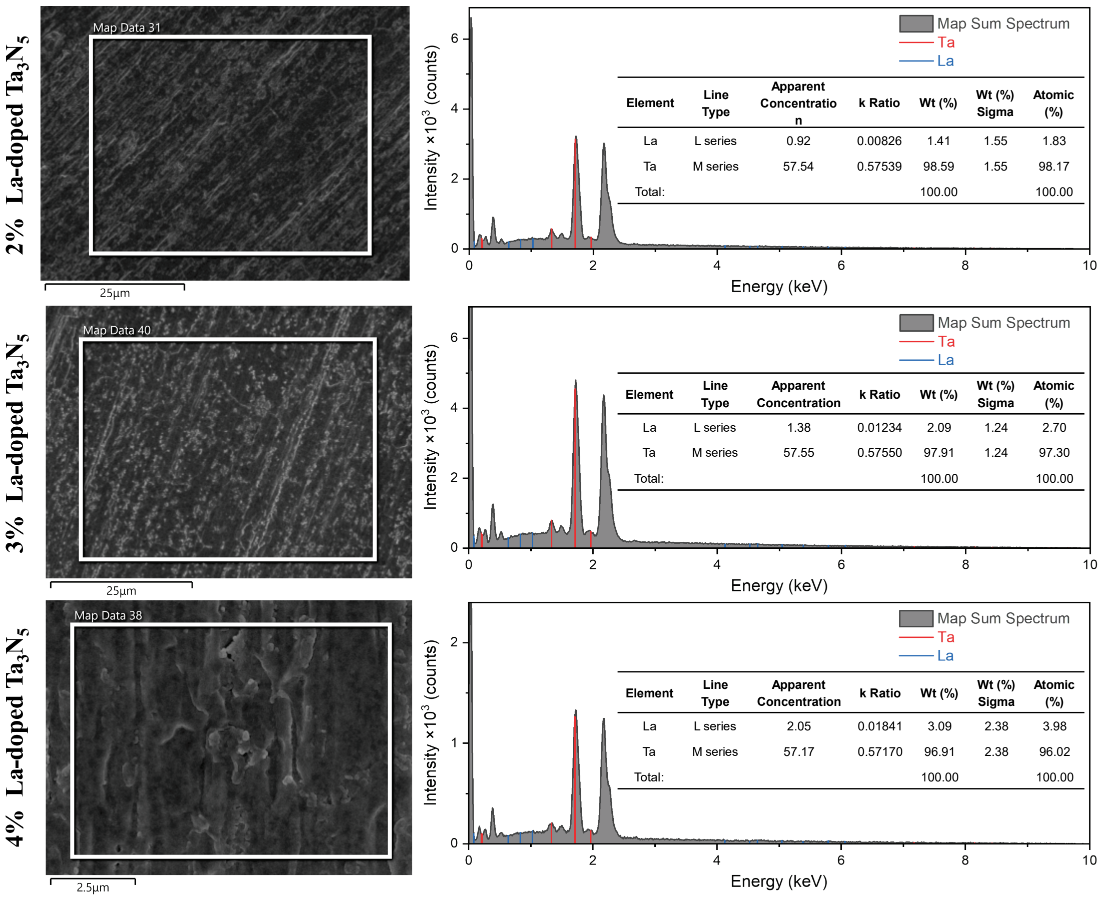

Element	Line Type	Apparent Concentration	k Ratio	Wt (%)	Wt (%) Sigma	Atomic (%)
La	L series	0.92	0.00826	1.41	1.55	1.83
Ta	M series	57.54	0.57539	98.59	1.55	98.17
Total:				100.00		100.00

Element	Line Type	Apparent Concentration	k Ratio	Wt (%)	Wt (%) Sigma	Atomic (%)
La	L series	1.38	0.01234	2.09	1.24	2.70
Ta	M series	57.55	0.57550	97.91	1.24	97.30
Total:				100.00		100.00

Element	Line Type	Apparent Concentration	k Ratio	Wt (%)	Wt (%) Sigma	Atomic (%)
La	L series	2.05	0.01841	3.09	2.38	3.98
Ta	M series	57.17	0.57170	96.91	2.38	96.02
Total:				100.00		100.00

Supplementary Fig. 11 | EDS analysis of La and Ta in Ta_3N_5 films with different La doping concentrations. The tables in the inset show the corresponding quantitative EDS analysis results.

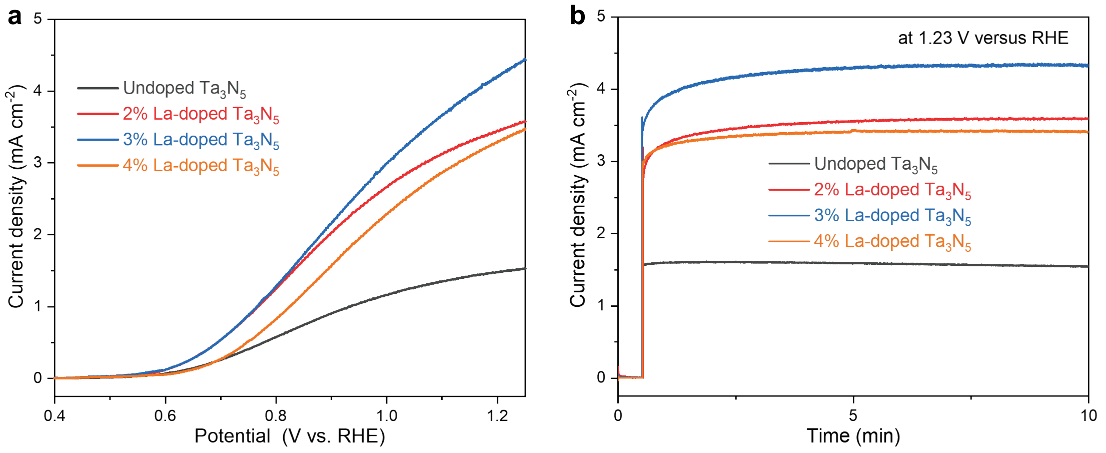

Supplementary Fig. 12 | PEC performance of Ta_3N_5 thin film (100 nm thick) photoanodes with different La doping concentrations. All the samples were modified with NiCoFe-B_i cocatalyst and tested under AM 1.5G simulated sunlight in 1 M KOH. **a**, *J*-V curves. **b**, Steady-state photocurrent of the photoanodes at an applied potential of 1.23 V versus RHE for 10 min.

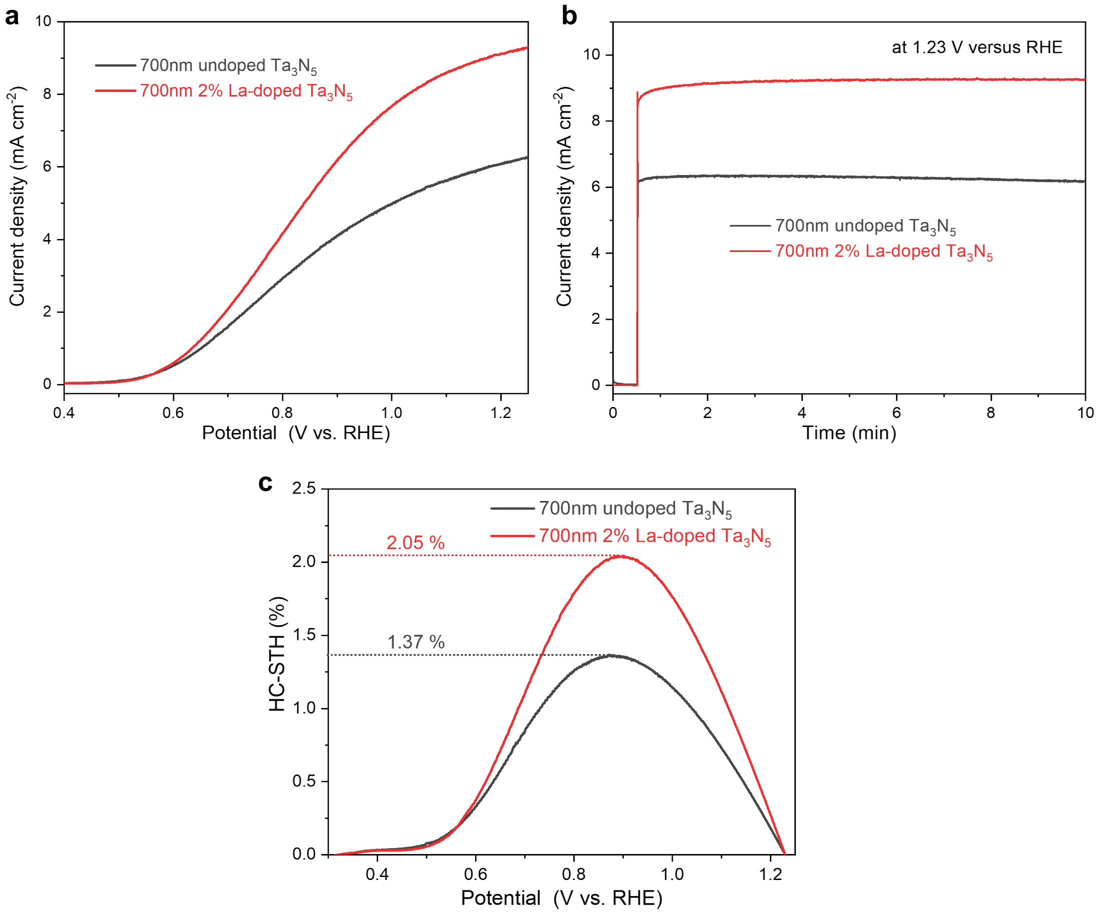

Supplementary Fig. 13 | PEC performance of undoped and 2% La-doped Ta_3N_5 thin film (700 nm thick) photoanodes. Both samples were modified with NiCoFe-B_i cocatalyst and tested under AM 1.5G simulated sunlight in 1 M KOH. **a**, *J*-V curves. **b**, Steady-state photocurrent at an applied potential of 1.23 V versus RHE for 10 min. **c**, HC-STH of the photoanodes calculated from *J*-*V* curves in (**a**).

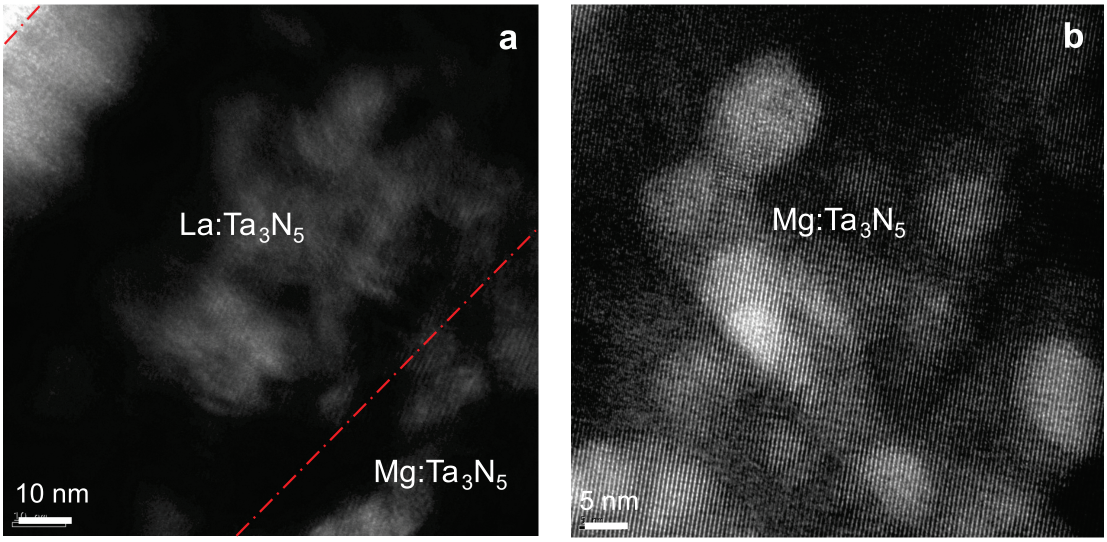

Supplementary Fig. 14 | HRTEM images of the gradient-Mg:Ta_3N_5/La:Ta_3N_5 film on Nb substrate. **a**, Surface La:Ta_3N_5 layer. **b**, Bulk gradient-Mg:Ta_3N_5 layer.

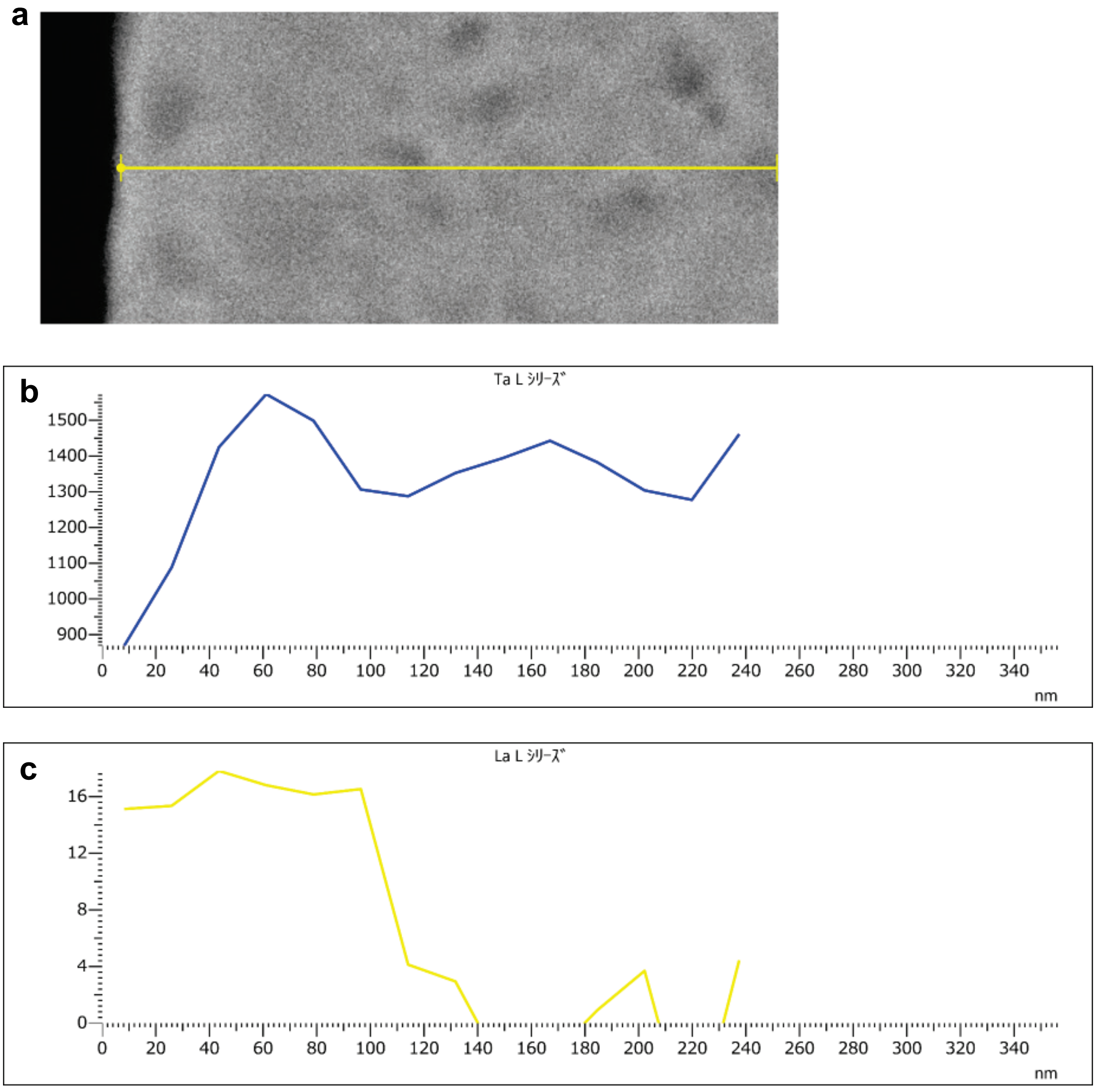

Supplementary Fig. 15 | STEM-EDS line scans in the surface region of the gradient-Mg:Ta_3N_5/La:Ta_3N_5 film. STEM image (**a**) and corresponding line scans for Ta (**b**) and La (**c**) elements.

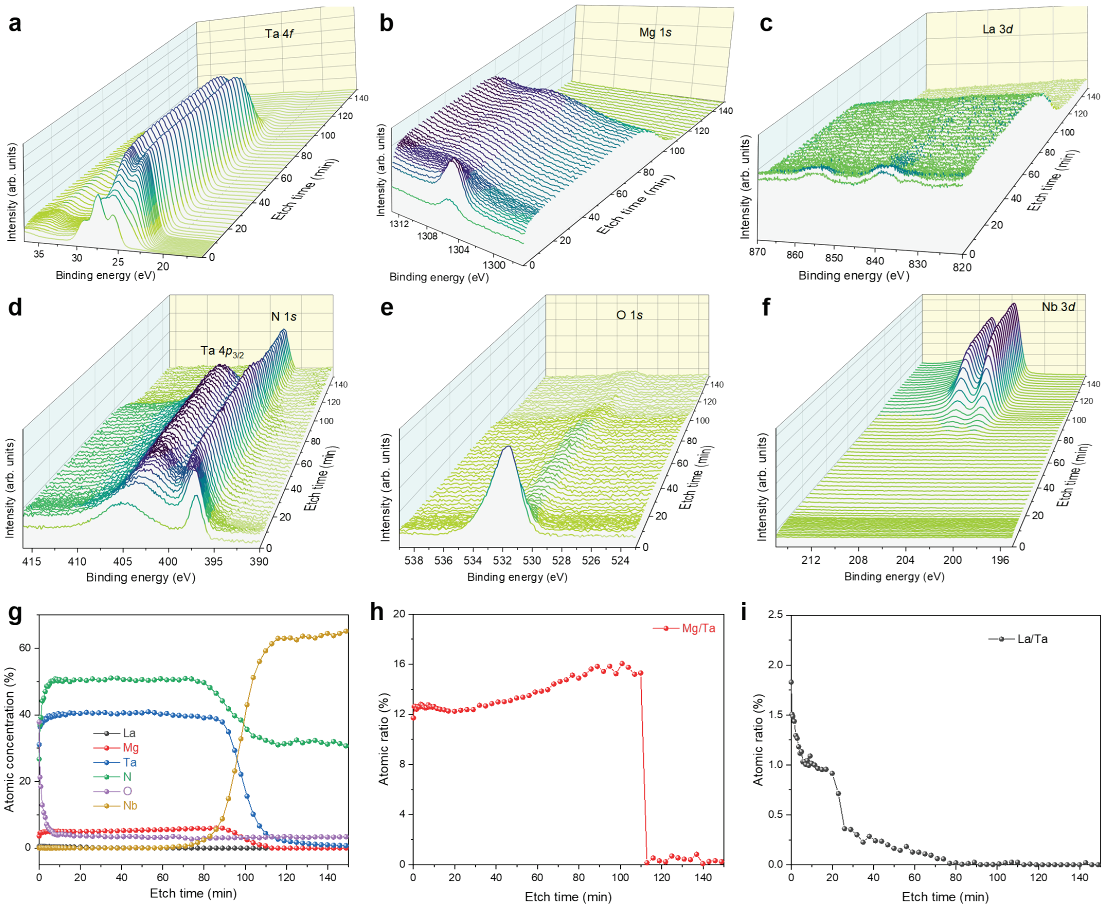

Supplementary Fig. 16 | XPS depth profile for gradient-Mg:Ta_3N_5/La:Ta_3N_5 film on Nb substrate. a-f, Ta 4*f*, Mg 1*s*, La 3*d*, N 1*s*, O 1*s,* and Nb 3*d* peaks as a function of etching time, respectively. **g**, XPS depth profile analyses of elemental concentrations. **h-i**, The change of atom ratios of Mg/Ta and La/Ta in the film with etching time. In the high binding energy region of Mg 1*s*, surface damage due to ion etching results in a decrease in peak intensity accompanied by an increase in the background level on the high binding energy side (*Ref. 2*). In order to make a more reliable quantitative analysis, a physically more meaningful Tougard-type background subtraction method was employed for peak fitting of the XPS peaks of Mg 1*s*. The Tougard-type background takes into account the inelastic scattering of electrons and has been widely used in quantitative XPS analysis (*Ref. 3*).

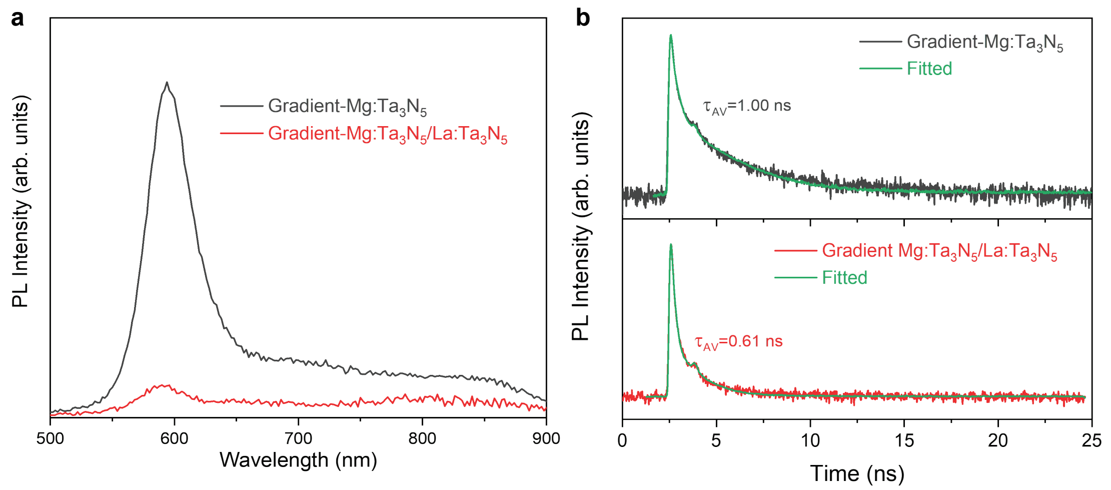

Supplementary Fig. 17 | Room-temperature PL spectra (a) and TRPL curves (b) of gradient-Mg:Ta_3N_5 and gradient-Mg:Ta_3N_5/La:Ta_3N_5 films on quartz substrates. A 420 nm laser was used as an excitation source for room temperature PL measurements. TRPL curves were acquired for the near-band edge emission at about 590 nm with 20 nm bandwidth under the excitation of a 420 nm picosecond laser pulsed at repetition rate of 40 MHz. Both decay curves were fitted using a biexponential decay model. The intensity-weighted average lifetime (τ_{AV}) is calculated by: $\tau_{AV} = f_1\tau_1 + f_2\tau_2$, where f_1 and f_2 are the fractional intensities of the decay channels with lifetimes τ_1 and τ_2, respectively. The lifetimes of the fast decay component (τ_1) and the slow component (τ_2) for the gradiet-Mg:Ta_3N_5 film were 0.25 ns and 2.00 ns, and the fractional intensities were 57.2% and 42.8%, respectively. For the gradiet-Mg:Ta_3N_5/La:Ta_3N_5 film, τ_1 and τ_2 were 0.14 ns and 1.22 ns, and the fractional intensities were 56.5% and 43.5%, respectively.

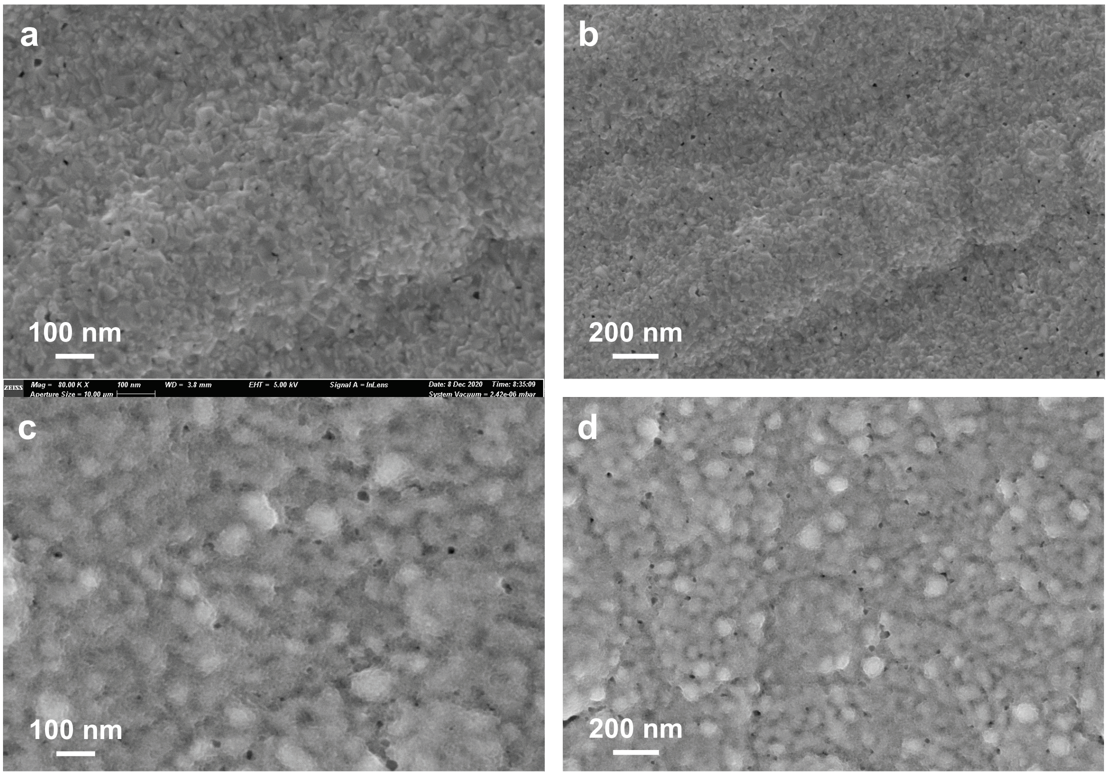

Supplementary Fig. 18 | Top-view SEM images of a gradient-$Mg:Ta_3N_5$/$La:Ta_3N_5$ film before (**a, b**) and after (**c, d**) NiCoFe-B_i cocatalyst modification.

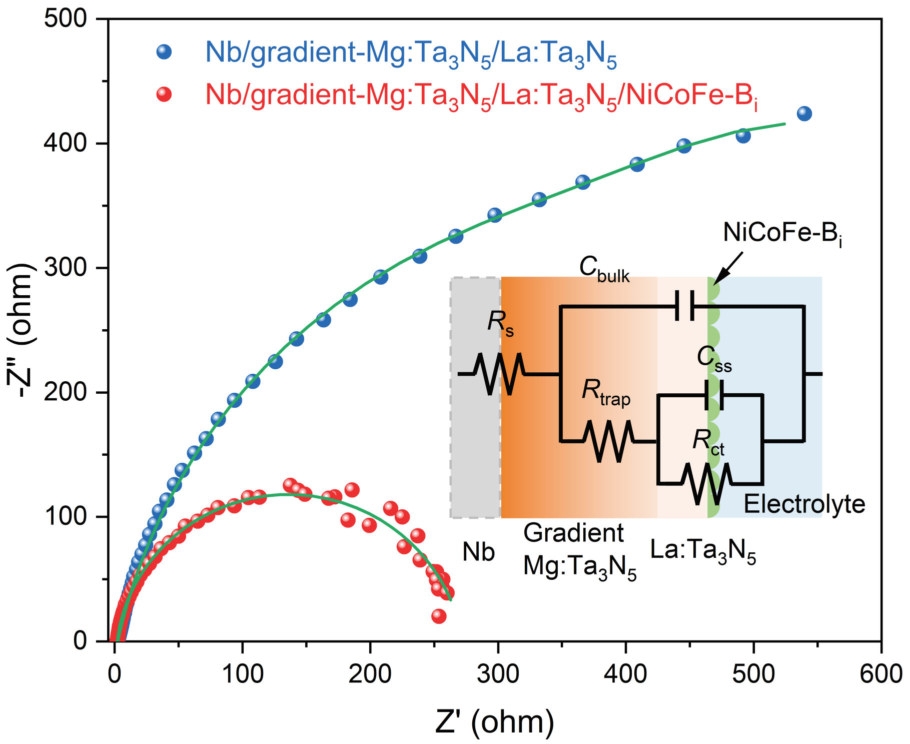

Supplementary Fig. 19 | Photoelectrochemical impedance spectroscopy (PEIS) for the gradient-Mg:Ta_3N_5/La:Ta_3N_5 photoanode with/without NiCoFe-B_i cocatalyst modification. The PEIS data were measured in 1 M KOH electrolyte at 1.0 V versus RHE under AM 1.5G simulated sunlight. Green lines denote the fitting of the PEIS data. The inset shows a two-RC-unit equivalent circuit used to fit the Nyquist plot of the PEIS, which consists of three resistances and two capacitors: a series resistance (R_s) of the electrolyte, external contact, and conductive substrate layer, a bulk charge transport resistance (R_{trap}), a semiconductor/electrolyte charge transfer resistance (R_{ct}), a bulk capacitor of space charge region (C_{bulk}), and a surface states capacitor (C_{ss}). The photoanode without cocatalyst modification showed a larger semicircle diameter, while the photoanode with the cocatalyst modification showed a smaller semicircle diameter, indicating that the charge transfer resistance of the photoanode was reduced after the NiCoFe-B_i cocatalyst modification. The fitted values of R_s, R_{trap} and R_{ct} from the equivalent circuit are displayed in Supplementary Table 4.

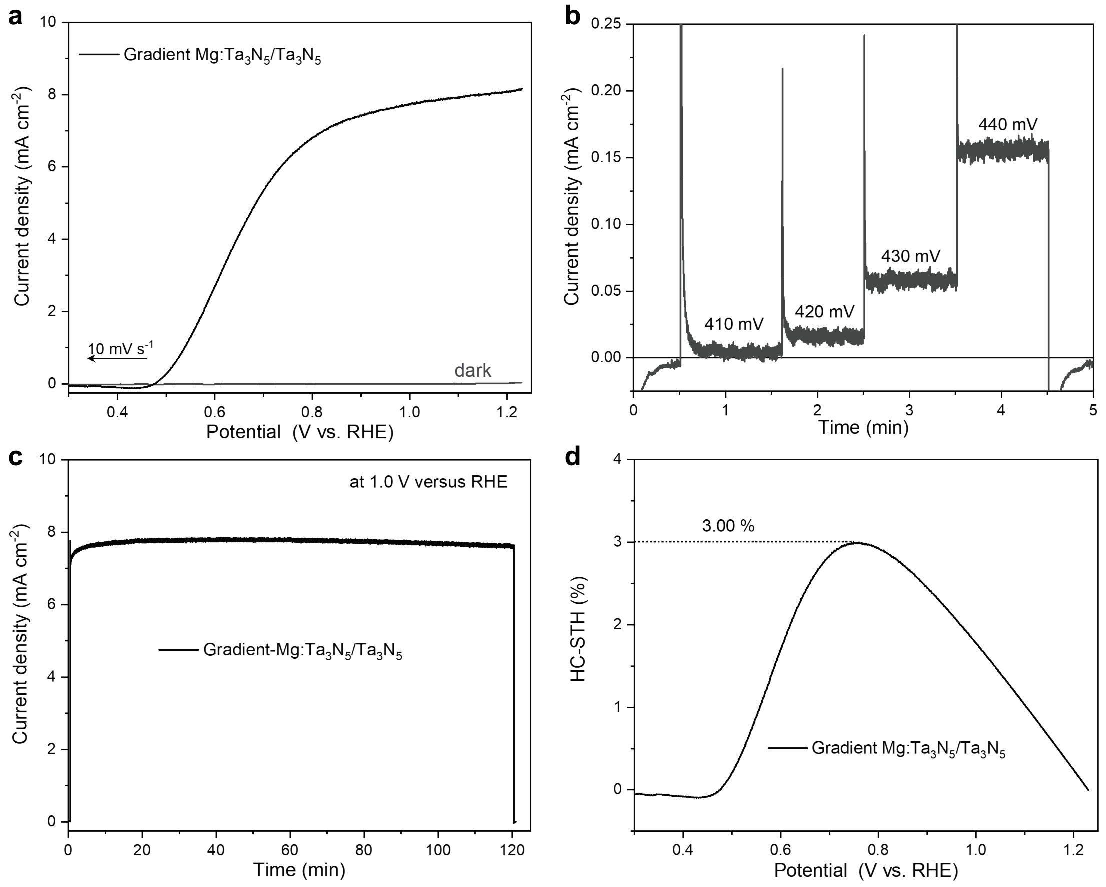

Supplementary Fig. 20 | Solar-driven PEC water oxidation properties of gradient-Mg:Ta_3N_5/Ta_3N_5 modified with NiCoFe-B_i cocatalysts. a, *J*-V curves under AM 1.5G simulated sunlight in 1 M KOH. **b**, Steady-state photocurrent of gradient-Mg:Ta_3N_5/Ta_3N_5 photoanode under low applied potentials. **c**, Steady-state photocurrent at an applied potential of 1.0 V versus RHE for 120 min. **d**, HC-STH of the photoanodes calculated from *J-V* curves in (**a**). The surface undoped Ta_3N_5 layer in the gradient-Mg:Ta_3N_5/Ta_3N_5 film has the same thickness as the La-doped Ta_3N_5 layer in the gradient-Mg:Ta_3N_5/La:Ta_3N_5 film. The gradient-Mg:Ta_3N_5/Ta_3N_5 photoanode showed a decreased photocurrent density of 8.2 mA cm^{-2} at 1.23 V versus RHE, indicating that the increased photocurrent of gradient-Mg:Ta_3N_5/La:Ta_3N_5, compared with gradient-Mg:Ta_3N_5, was not due to an increase in the thickness of the photoanode. A steady photocurrent density of ~20 μA cm^{-2} was generated at 0.42 V versus RHE, revealing that the introduction of an undoped layer of Ta_3N_5 resulted in a positive shift of onset potential for the gradient Mg:Ta_3N_5/Ta_3N_5 photoanode.

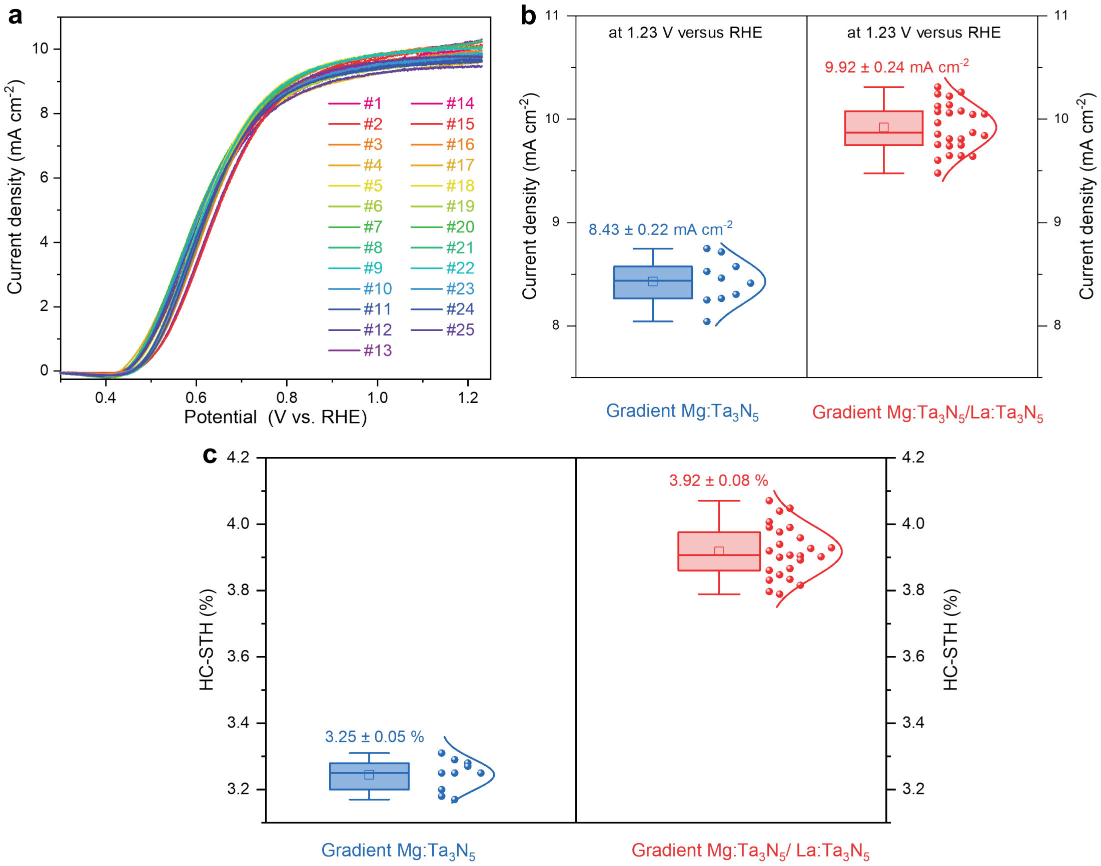

Supplementary Fig. 21 | Reproducibility of the PEC performance for gradient-Mg:Ta_3N_5/La:Ta_3N_5 photoanodes. All samples were modified with NiCoFe-B_i cocatalyst and tested under AM 1.5G simulated sunlight in 1 M KOH. **a**, *J-V* curves for a batch of 25 gradient-Mg:Ta_3N_5/La:Ta_3N_5 photoanodes. **b**, Statistics of the photocurrents for the gradient-Mg:Ta_3N_5/La:Ta_3N_5 photoanodes in (**a**) and gradient-Mg:Ta_3N_5 photoanodes (data extracted from *Ref. 1*) at 1.23 V versus RHE. **c**, Statistics of the HC-STHs for the gradient-Mg:Ta_3N_5/La:Ta_3N_5 photoanodes in (**a**) and gradient-Mg:Ta_3N_5 photoanodes (data extracted from *Ref. 1*).

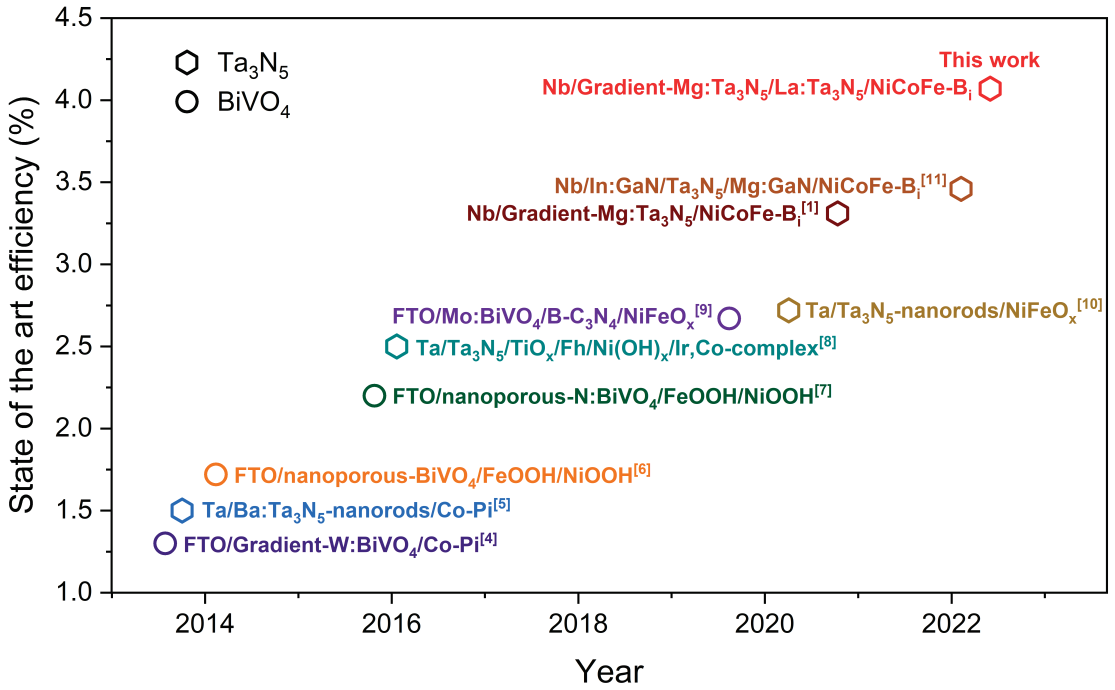

Supplementary Fig. 22 | Reported state-of-the-art HC-STH for photoanodes based on $BiVO_4$ and Ta_3N_5. The HC-STH values are extracted from Supplementary *Ref. 1, 4-11*.

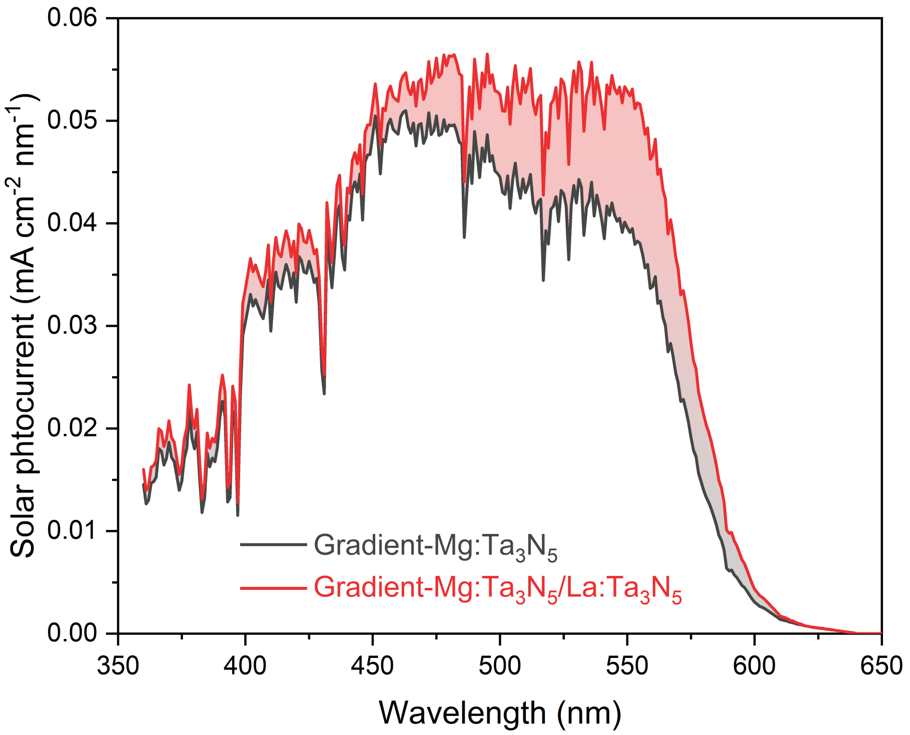

Supplementary Fig. 23 | The solar photocurrent spectra for gradient-Mg:Ta_3N_5 and gradient-Mg:Ta_3N_5/La:Ta_3N_5 photoanodes. The solar photocurrent spectra were calculated by multiplying the IPCE spectra (Fig. 5e) with the standard AM 1.5G spectrum (ASTM G173-03).

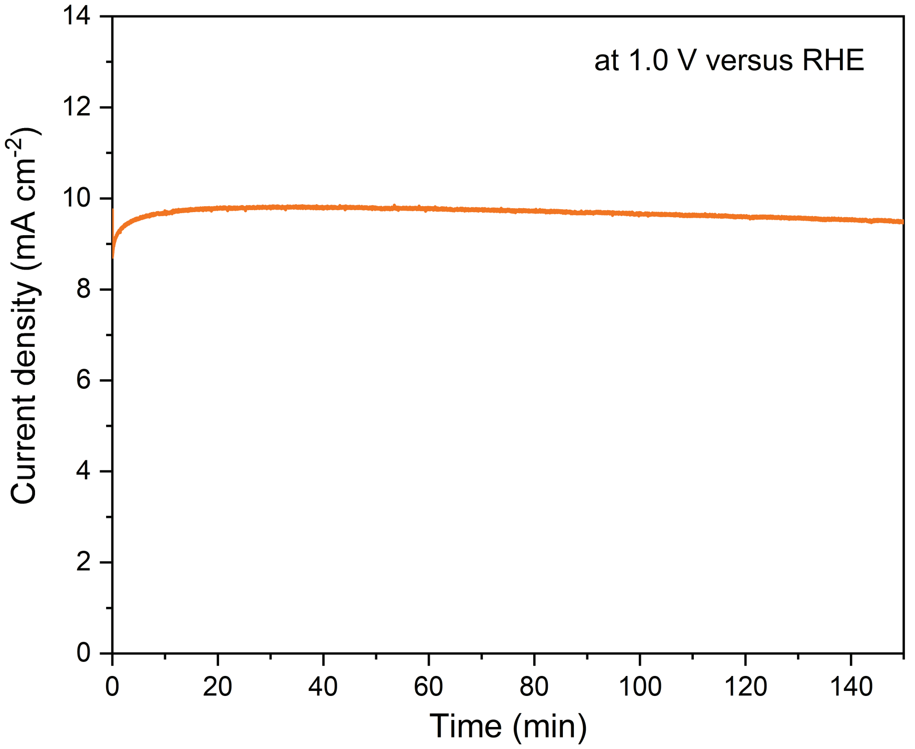

Supplementary Fig. 24 | Photocurrent recorded during the gas chromatography measurement. The gradient-Mg:Ta_3N_5/La:Ta_3N_5 photoanode was modified with NiCoFe-B_i cocatalysts and tested at 1.0 V vs. RHE in 1 M KOH under AM 1.5G simulated sunlight for 150 min.

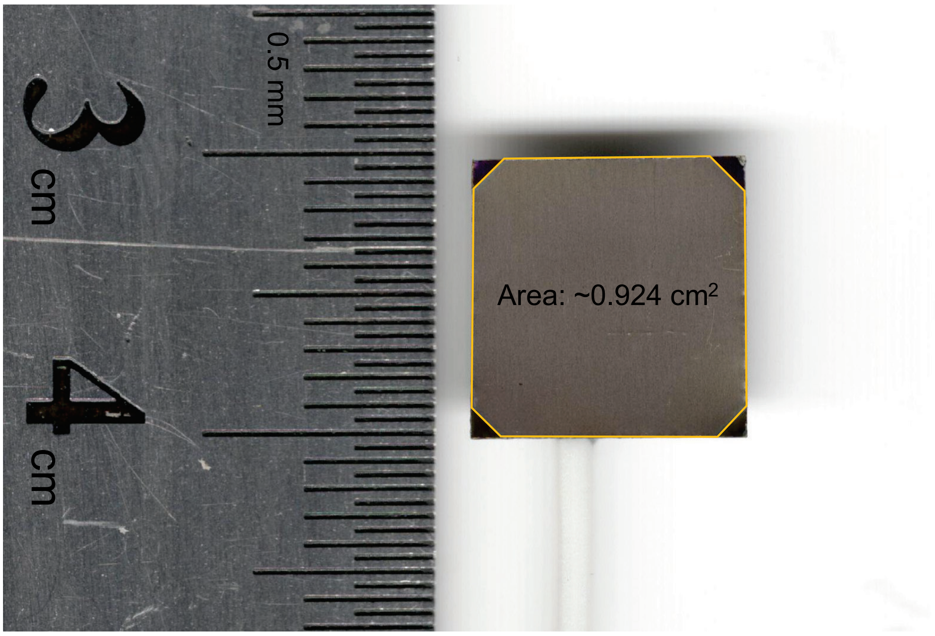

Supplementary Fig. 25 | Photographic image of fabricated photoanode. The exposed area of the electrode was measured with ImageJ[1.53k] software.

Supplementary Table 1 | The detailed parameters of the *J-V* curves for Ta_3N_5-based photoanodes in this study. The onset potential is defined as the potential at a photocurrent density of 0.1 mA cm^{-2}. For heterogeneous doped Ta_3N_5 photoanodes, the steady-state photocurrent at low bias conditions was measured to determine the onset potential.

Doping level	Thickness (nm)	Onset potential (V versus RHE)	Photocurrent at 1.23 V versus RHE (mA cm^{-2})	HC-STH (%)
**Undoped and La-doped Ta_3N_5 photoanodes**				
Undoped	100	0.63	1.51	0.30
	300	0.58	3.68	0.93
	500	0.55	5.32	1.06
	700	0.50	6.18	1.37
2%	100	0.59	3.52	0.67
	700	0.51	9.23	2.05
3%	100	0.58	4.40	0.80
	300	0.57	5.04	0.87
	500	0.58	4.02	0.60
	700	0.59	3.77	0.52
4%	100	0.64	3.41	0.55
**Heterogeneous doped Ta_3N_5 photoanodes**				
Gradient-Mg:Ta_3N_5		0.39	8.51	3.30
Gradient-Mg:Ta_3N_5/Ta_3N_5		0.42	8.17	3.00
Gradient-Mg:Ta_3N_5/La:Ta_3N_5		0.39	10.06	4.07

Supplementary Table 2 | The crystal grain size in the undoped and La-doped Ta_3N_5 films calculated by applying the Scherrer Equation to the (110) peak near 24.5° and the (203) peak near 31.5°.

**Peak position**	**Crystallite size (nm)**			
	Undoped Ta_3N_5	2% La-doped Ta_3N_5	3% La-doped Ta_3N_5	4% La-doped Ta_3N_5
(110) 24.5°	44.9	37.1	31.6	22.9
(203) 31.5°	50.6	44.9	38.0	25.6

Supplementary Table 3 | Effective masses of electron and hole in the Brillouin zone of undoped and La-doped Ta_3N_5 along different directions in units of the electron rest mass (m_0).

Sample	Electron			Hole	
	Γ → X	Y → Γ	Γ → Z	Γ → X	X → S
Undoped Ta_3N_5	0.50	7.73	1.85	1.68	1.75
La-doped Ta_3N_5	0.65	10.84	1.96	2.67	1.76

Supplementary Table 4 | Fitted values of R_s, R_{trap}, and R_{ct} of the gradient-Mg:Ta_3N_5/La:Ta_3N_5 photoanode with/without NiCoFe-B_i cocatalyst modification.

**Photoanodes**	**R_s (Ohm)**	**R_{trap} (Ohm)**	**R_{ct} (Ohm)**
Gradient-Mg:Ta_3N_5/La:Ta_3N_5	2.00	3.18	913.9
Gradient-Mg:Ta_3N_5/La:Ta_3N_5/NiCoFe-B_i	1.89	2.09	268.3

Supplementary References:

1. Xiao, Y. *et al.* Band structure engineering and defect control of Ta_3N_5 for efficient photoelectrochemical water oxidation. *Nat. Catal.* **3**, 932-940 (2020).
2. Greczynski, G. & Hultman, L. X-ray photoelectron spectroscopy: Towards reliable binding energy referencing. *Prog. Mater Sci.* **107**, 100591 (2020).
3. Tougaard S. Practical guide to the use of backgrounds in quantitative XPS. *J. Vac. Sci. Technol. A* **39**, 011201 (2021)
4. Abdi, F. F. *et al.* Efficient solar water splitting by enhanced charge separation in a bismuth vanadate-silicon tandem photoelectrode. *Nat. Commun.* **4**, 2195 (2013).
5. Li, Y. *et al.* Cobalt phosphate-modified barium-doped tantalum nitride nanorod photoanode with 1.5% solar energy conversion efficiency. *Nat. Commun.* **4**, 2566 (2013).
6. Kim, T. W. *et al.* Nanoporous $BiVO_4$ photoanodes with dual-layer oxygen evolution catalysts for solar water splitting. *Science* **343**, 990-994 (2014).
7. Kim, T. W. *et al.* Simultaneous enhancements in photon absorption and charge transport of bismuth vanadate photoanodes for solar water splitting. *Nat. Commun.* **6**, 8769 (2015).
8. Liu, G. *et al.* Enabling an integrated tantalum nitride photoanode to approach the theoretical photocurrent limit for solar water splitting. *Energy Environ. Sci.* **9**, 1327-1334 (2016).
9. Ye, K. H. *et al.* Enhancing photoelectrochemical water splitting by combining work function tuning and heterojunction engineering. *Nat. Commun.* **10**, 3687 (2019).
10. Pihosh, Y. *et al.* Ta_3N_5-Nanorods enabling highly efficient water oxidation via advantageous light harvesting and charge collection. *Energy Environ. Sci.* **13**, 1519-1530 (2020).
11. Fu, J. *et al.* Interface engineering of Ta_3N_5 thin film photoanode for highly efficient photoelectrochemical water splitting. *Nat. Commun.* **13**, 729 (2022).
